# Patterns of progressive atrophy vary with age in Alzheimer's disease patients

**DOI:** 10.1016/j.neurobiolaging.2017.11.002

**Published:** 2018-03

**Authors:** Cassidy M. Fiford, Gerard R. Ridgway, David M. Cash, Marc Modat, Jennifer Nicholas, Emily N. Manning, Ian B. Malone, Geert Jan Biessels, Sebastien Ourselin, Owen T. Carmichael, M. Jorge Cardoso, Josephine Barnes

**Affiliations:** a Dementia Research Centre, Department of Neurodegenerative Disease, UCL Institute of Neurology, London, UK; b FMRIB Centre, Nuffield Department of Clinical Neurosciences, University of Oxford, Oxford, UK; c Wellcome Trust Centre for Neuroimaging, 12 Queen Square, London, UK; d Translational Imaging Group, Centre for Medical Image Computing, University College London, London, UK; e London School of Hygiene and Tropical Medicine, London, UK; f Department of Neurology and Neurosurgery, Brain Center Rudolf Magnus, University Medical Center Utrecht, Utrecht, the Netherlands; g Pennington Biomedical Research Center, Baton Rouge, LA, USA

**Keywords:** Aging, Early-onset Alzheimer's disease, Alzheimer's disease, Atrophy, Late-onset, Mild cognitive impairment (MCI), Hippocampus

## Abstract

Age is not only the greatest risk factor for Alzheimer's disease (AD) but also a key modifier of disease presentation and progression. Here, we investigate how longitudinal atrophy patterns vary with age in mild cognitive impairment (MCI) and AD. Data comprised serial longitudinal 1.5-T magnetic resonance imaging scans from 153 AD, 339 MCI, and 191 control subjects. Voxel-wise maps of longitudinal volume change were obtained and aligned across subjects. Local volume change was then modeled in terms of diagnostic group and an interaction between group and age, adjusted for total intracranial volume, white-matter hyperintensity volume, and apolipoprotein E genotype. Results were significant at *p* < 0.05 with family-wise error correction for multiple comparisons. An age-by-group interaction revealed that younger AD patients had significantly faster atrophy rates in the bilateral precuneus, parietal, and superior temporal lobes. These results suggest younger AD patients have predominantly posterior progressive atrophy, unexplained by white-matter hyperintensity, apolipoprotein E, or total intracranial volume. Clinical trials may benefit from adapting outcome measures for patient groups with lower average ages, to capture progressive atrophy in posterior cortices.

## 1 Introduction

Age is the largest risk factor for Alzheimer's disease (AD) (Launer et al., 1999). However, AD can develop at any point during the adult life span as early as the fourth decade. Patients and their carers need clear and accurate information regarding the presentation and progression of AD; therefore, investigating how disease progression varies with age is important for clinicians and health care professionals. Understanding the neurobiology of underlying age effects may also result in different markers for diagnosis and disease tracking for different age ranges. Finally, age differences in AD may impact the recruitment strategy for clinical trials and the choice of trial outcome measures.

The course of AD appears different in older versus younger AD subjects. In the clinic, younger AD patients are more likely to have nonmemory cognitive symptoms such as difficulties in language processing, attention, and visuospatial abilities (Barnes et al., 2015). About one-third of early-onset AD patients (typically defined as aged less than 65 years) have nonamnestic presentation, compared with 6% of late-onset AD patients (Koedam et al., 2010). Single time point studies reveal smaller volumes in association cortices such as the precuneus and posterior parietal cortex in younger compared with older patients; older patients have been found to have lower gray matter (GM) volumes in the medial temporal lobe (Frisoni et al., 2005, Möller et al., 2013). Neuropathological subtypes of dementia have been proposed, which divide atypical individuals into hippocampal-sparing AD and limbic-predominant AD, depending on the predominance of plaques and tangles in neocortical and medial temporal areas respectively (Murray et al., 2011); in vivo tissue loss in corresponding anatomical regions has been confirmed in these subtypes with ante-mortem magnetic resonance imaging (MRI) (Whitwell et al., 2012). Previous studies investigating longitudinal atrophy differences with age have found differences with respect to age, although being small in scale (Cho et al., 2013a), lacking adjustment for normal aging effects on atrophy within patient groups (Cho et al., 2013a, Hua et al., 2010), or having used regions of interest that are more associated with typical patterns of AD, and thus may not reflect the anatomical differences more often found in younger cases (Holland et al., 2012, Nosheny et al., 2015). A further article by Holland et al. found numerous regions more atrophic at younger ages in AD, including the medial temporal lobe and the inferior parietal lobe (Holland et al., 2013).

AD pathology is not the only driver of brain atrophy with age; there are other biological reasons which may explain why AD differs with age. Although baseline age may act as a potential marker of AD heterogeneity at younger ages, aging, or the accrual of damaging processes with time, also results in cerebral atrophy. Vascular disease is an important and potentially modifiable determinant of brain aging (Viswanathan et al., 2009). One measure of vascular disease burden is the volume of white-matter hyperintensities (WMHs) of presumed vascular origin observed on MRIs, which increase with age and are associated with subsequent atrophy and cognitive decline (Barnes et al., 2013, Kloppenborg et al., 2012, Schmidt et al., 2005, Schmidt et al., 2011). Genes also influence AD: the apolipoprotein E (APOE) e4 allele is another important risk factor for atrophy patterns and age at onset; it is implicated in an earlier age at onset (Breitner et al., 1998, Corder et al., 1993), targeted hippocampal atrophy (Manning et al., 2014), and although it is a risk factor for 1 presentation of atypical early-onset AD: posterior cortical atrophy (PCA) (Schott et al., 2015), its effects are weaker than for typical AD.

In this study, we investigated the effect of age on atrophy rate patterns in a large multisite data set of predominantly late-onset MCI and AD patients allowing for normal aging effects, WMH volume, and APOE e4 status. We used a novel multi-time point voxel-wise technique to investigate the age effects on brain atrophy over time without a priori anatomical hypotheses. We also looked to see if any differences with age remained in a cohort of individuals with confirmed underlying AD pathology from cerebrospinal fluid (CSF) data. The longitudinal registration and statistical modeling techniques used in this study allowed voxel-wise change across the brain to be studied while allowing for missing data, therefore enabling inclusion of patients with at least 1 follow-up scan, who dropout before the end of the study. This is important in the context of age, as it has been shown that dropout is unlikely to be random and may be due to higher levels of frailty, poorer vascular health, or rapid decline (Fiford et al., 2017, Manning et al., 2017). Whole brain and hippocampal boundary shift integral (BSI) results were used to supplement voxel-wise findings (Leung et al., 2012). We hypothesized that AD patients with a younger age at baseline would show more widespread cortical atrophy relative to controls than older patients, which would not be accounted for by APOE e4 carrier status or vascular disease as measured by WMH burden. We further hypothesized that the MCI group would show similar but less extensive effects.

To compare younger and older AD subjects while accounting for normal aging, which may incur additional tissue loss for older AD subjects, we built a model to predict atrophy rate with an interaction term between disease group and baseline age. The interaction term allows the effect of age on atrophy rate in controls to be subtracted from the slope of age and atrophy rate in patient groups. This approach shows the relationship of baseline age on atrophy rate in patient groups after accounting for normal aging.

## 2 Methods

### 2.1 Participants

All study data were obtained from the Alzheimer’s disease neuroimaging initiative (ADNI) database (adni.loni.usc.edu). Participants took part in baseline clinical, neuropsychometric and MRI assessments, and periodical assessments thereafter. Written informed consent was approved by the institutional review board at each of the >50 participating centers. ADNI is a multicentre longitudinal private-publicly funded study launched in 2003 investigating healthy controls, MCI, and AD subjects. Based in USA, ADNI is headed by Michael W. Weiner. The principle goal of ADNI has been to test whether serial MRI, positron emission tomography (PET), biomarkers, and clinical and neuropsychological data usage could measure the progression of MCI and early AD. For up to date information please see www.adni-info.org.

All MCI and AD patients were required to have a memory complaint confirmed by a study partner and abnormal education-adjusted score on the Logical Memory II (Delayed Paragraph Recall) from the Wechsler Memory Scale.

### 2.2 Image acquisition and assessment

The ADNI MRI protocol is detailed elsewhere (Jack et al., 2008). After the acquisition, quality control was completed at the Mayo clinic (Rochester, MN, USA) including a protocol compliance check, image quality control, and inspection for clinically significant medical abnormalities. Standard ADNI image preprocessing was then applied, including gradient warping (Jovicich et al., 2006), B1 nonuniformity (Narayana et al., 1988), and intensity nonuniformity correction (Sled et al., 1998). In addition, internal visual quality control checking was performed before analysis, excluding images with significant motion artifacts resulting in severe blurring of tissue boundaries.

### 2.3 Statistics: demographics and baseline volumetrics

WMH volumes were downloaded from the ADNI Laboratory of Neuro Imaging Image Data Archive (http://adni.loni.usc.edu/), which were previously segmented using an automated technique from baseline PD, T1, and T2 weighted images (Carmichael et al., 2010, Schwarz et al., 2009). These volumes were log-transformed (base 2) to reduce skewness. Linear regression analyses, with F tests, were used to test for between-group (control, MCI, and AD) differences in baseline age, Mini–Mental State Examination (MMSE), total intracranial volume (TIV), WMH, whole-brain and hippocampal volume. Fisher's exact test was used to investigate gender differences. For WMH, brain and hippocampal volume analyses, TIV was added as a covariate. TIVs were calculated using a previously described SPM12b-based automated technique (Malone et al., 2015).

### 2.4 Image analysis: longitudinal voxel-based morphometry (VBM)

Imaging data consisted of all available ADNI1 time points from baseline to 36 months (0-, 6-, 12, 18-, 24-, and 36- month scans), where a T1-weighted volumetric scan acquired on a 1.5-T scanner was available and of sufficient quality. Using the serial longitudinal registration tool in SPM12, all scans for a given subject were nonlinearly registered to a within-subject space unique to that individual, incorporating a bias field correction accounting for any differences in image inhomogeneities between scans (Ashburner and Ridgway, 2012). Resultantly, an unbiased average image was produced corresponding to a midpoint between all time points for that person. From the nonlinear registrations, Jacobian images of the rate of volumetric voxel expansion and contraction were additionally produced for each time point encoding the voxel change for each image with reference to the individual's midpoint image. These volume-change maps and within-subject averages were visually checked for registration errors.

The midpoint average images were segmented into GM and WM, then registered with DARTEL (Ashburner, 2007), which nonlinearly registers the individuals to create a group-specific space based on the simultaneous alignment of each tissue type. GM, WM segments, and volume-change maps from the longitudinal registrations were then transformed to the group-wise space by applying the flow fields from the previous step.

Using the DARTEL-transformed GM and volume-change maps, tissue-weighted smoothing (also known as normalized convolution) was applied to smooth the volume-change maps with a Gaussian kernel (of 6 mm full width at half maximum) using only data within the limits of the tissue segments (binarized at 0.5). This produced a tissue-specific smoothed volume-change map in which each voxel's value represents the expansion or contraction of that tissue during serial longitudinal registration. These steps were repeated for WM. The resultant smoothed GM and WM tissue-weighted volume-change maps were then used for longitudinal analysis. Masks for analysis were made using the smoothed, modulated, DARTEL-warped segments. The technique used averages all segments and creates a mask based on optimal thresholding of the average, which maximizes the correlation between the original segment and the thresholded segment (Ridgway et al., 2009).

### 2.5 Image analysis: volumes and volume changes

Brain and hippocampal volumes were estimated automatically from the 1.5-T volumetric T1-weighted images using BMAPS (Leung et al., 2011 and HMAPS respectively (Leung et al., 2010), multi-atlas template segmentation methods. The BSI was used to estimate change directly from scan pairs following segmentation (Leung et al., 2012), the outcome representing change in volume of brain or hippocampus (mL) during the scan interval.

### 2.6 Image statistics: voxel-wise statistical analysis

Using the Sandwich Estimator toolbox (http://www.fil.ion.ucl.ac.uk/spm/ext/#SwE) in SPM, a marginal model was fitted to the voxel-wise volume-change data (Guillaume et al., 2014). The marginal model accounts for the intra-visit correlations, which exist longitudinally, as well as the unbalanced nature of the data; subjects have different numbers of time points due to study design and also subject dropout. Other mixed modeling methods exist for longitudinal imaging (Bernal-Rusiel et al., 2013, Li et al., 2013, Skup et al., 2012, Ziegler et al., 2015) but require iterative optimization at each voxel and sometimes fail to converge. In addition, these models require specification of the random effects and covariance structure of the error terms; such specifications are complex, and their misspecification can lead to invalid results. In the Sandwich Estimation method used, fixed effects only are estimated in the marginal model, and random components are modeled as nuisance; as such the random effects do not need to be specified (Guillaume, 2015).

For each volume-change map, the interval in years from the image to midpoint was included as a fixed effect in the model (time-from-midpoint), to model annualized voxel change as an outcome. All covariates were allowed to interact with time-from-midpoint, in order that their inclusion could influence the rate of change in volume. We used TIV, WMH, and APOE e4 as covariates. TIV was used as a proxy for maximal (premorbid) brain size, WMH was chosen to remove the effect of vascular disease, and APOE e4 genotype (a binary variable indicating possession of at least one e4 allele) was included as it has been known to influence patterns of atrophy and age at onset. We also constructed models without WMH and APOE e4 genotype to understand whether their exclusion influenced the relationship between baseline age and atrophy patterns.

Notably, age at baseline (age at first assessment in the study) was used as our measure of age. We chose to use baseline age as the passing of time during which the study was encoded in the interval variable (time-from-midpoint). Where age is mentioned throughout the text, we are referring to age at baseline.

The primary model investigated GM volume change (outcome) with a main effect of group, a linear interaction term between group and baseline age, and covariates of TIV, WMH, and APOE e4 genotype (model 1). The secondary model was constructed identically to the previous, omitting WMH and APOE e4 genotype, (model 2). These 2 models were repeated using the outcome of WM volume change in place of GM volume change (model 3, with WMH and APOE adjustment, and model 4 without WMH and APOE adjustment).

For each model, an F-contrast was applied to test the overall significance of the age-by-group interaction term across all groups. The age-by-group interaction term was also used to investigate pair-wise differences between groups using *t* tests. The first *t* test was applied to investigate whether the relationship between age and atrophy rate was significantly different in control and AD groups at each voxel, by calculating the difference in the age-by-group interaction term for these groups. Differences between control and MCI groups were subsequently explored.

Models were run using the wild bootstrap with 2000 iterations to obtain results corrected for multiple comparisons using family-wise error (FWE); results were then thresholded at *p* < 0.05.

For the model investigating GM volume change (model 1), individual summarized slopes of volume change were generated at specific voxels, which were then used to make graphs. Voxels were chosen within clusters which survived FWE correction, and summary slopes at that voxel were plotted against baseline age for each individual [for illustrative purposes, cf (Kriegeskorte et al., 2010)].

### 2.7 Image statistics: change in volumes

We fitted multilevel linear mixed-effects regression models for repeated measures of direct change, with the dependent variable BSI (mL of brain/hippocampal change during the scan interval) (Frost et al., 2004). Interval in years between baseline and follow-up was included as a fixed effect, in order that the resulting coefficient represented volume change in milliliter per year (outcome). The following covariates were included as interaction terms with interval, in order that their inclusion could affect atrophy rate: diagnostic group, an interaction between baseline age and diagnostic group, WMH, APOE e4 carrier status, and TIV. A participant level random effect for scan interval was included to permit between-participant heterogeneity in atrophy rate, with different random slope terms fitted for control, MCI, and AD groups, as the variability in atrophy rate is often higher in AD patients. For each diagnostic group, a different participant level random intercept term was included to allow for the correlation between BSI measures from the same baseline scan. No intercept was included in the model due to the assumption that the estimated atrophy rate over a scan interval of zero is zero.

After estimation, the difference in the age effect on atrophy rate for MCI/AD and the age effect on atrophy rate in controls was estimated to determine the increased or decreased atrophy rate with respect to normal aging. Models were fitted for the hippocampus and whole brain separately. To aid comprehension, the results of the age–diagnostic group interaction for each diagnostic group are given as the effect of age on atrophy rates, rather than relative to controls, although the difference versus controls was tested to see if there was an MCI or AD specific effect of age.

### 2.8 Further analyses

We also tested whether the relationship between age and atrophy rates remained in a subset with confirmed amyloid pathology from CSF data. Voxel-wise and region of interest (ROI) analyses were repeated as aforementioned (Sections 2.6, 2.7). See Supplementary Material for more information on subject selection, methods, and results.

To investigate whether disease severity differed according to age, we looked at the effect of age on baseline brain volume. Details of this analysis and results can be found in the Supplementary Material.

To explore whether age affected cognitive decline, we investigated whether age predicted change in MMSE using linear mixed-effects models, see Supplementary Material for methods and results.

To assess the validity of using baseline age as a proxy for age at onset (estimated by the study partner), analyses were run to investigate GM volume change with (1) baseline age and (2) age at onset as predictors. These were then visually compared. See Supplementary Material for detailed information and results.

To test for nonlinearity in the age–atrophy relationship, a quadratic term was added to the models of GM change, see Supplementary Material for information and results.

## 3 Results

### 3.1 Group demographics

**Fig. 1:**
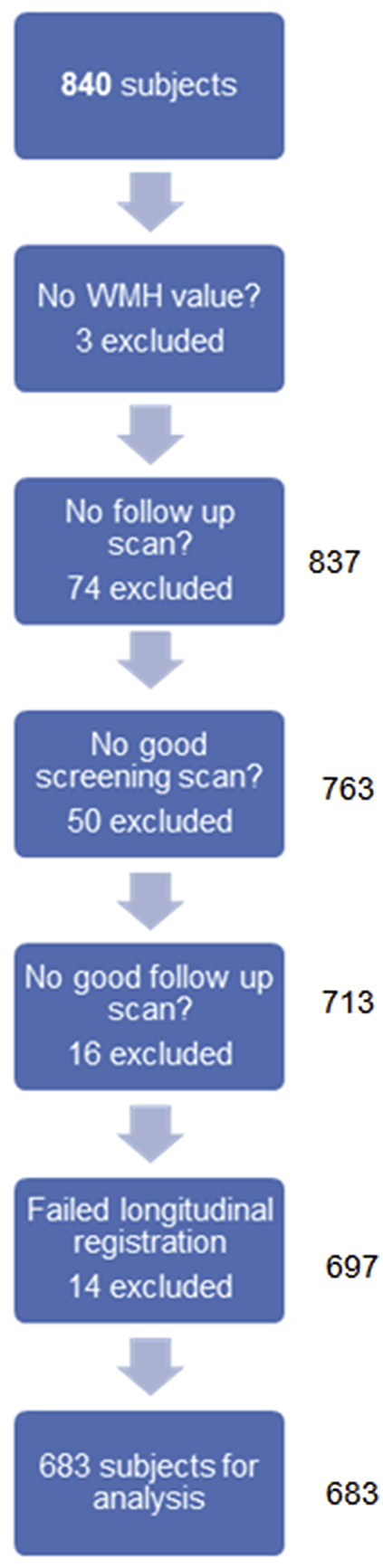
Flowchart showing the selection of subjects for analysis. Abbreviation: WMH, white-matter hyperintensity.

Data from 840 participants were downloaded from the ADNI website. Following quality control (see Section 2.2), 143 subjects were excluded (see Fig. 1); of which, 22% were controls, 34% had a diagnosis of MCI and 27% were diagnosed with AD, and 17% had no diagnostic information available (scans without diagnostic information were failed at initial visit by Laboratory of Neuro Imaging). After longitudinal registration (Section 2.4), 14 further subjects were dropped due to registration errors (7 controls, 6 individuals with MCI, and 1 with AD).

A total of 683 participants were included in this study, 191 controls, 339 individuals with MCI, and 153 individuals with AD passed quality control; amounting to 2972 images, see Table 1 for demographic and imaging information. Subjects differed in baseline MMSE, baseline brain and hippocampal volume, and APOE e4 genotype in a manner consistent with a diagnosis of MCI or AD. There were significantly more males in the MCI group, which likely explains the observed difference in TIV size between groups. For subject demographics split by mean age in each diagnostic group, see Supplementary Table 1.

**Table 1:** Subject demographics and basic imaging information

	Controls	MCI	AD	*p*-value across groups
N	191	339	153	
Age at baseline, y	75.9 (5.2)	75.0 (7.2)	75.0 (7.7)	0.3
Percentage male	51.8	62.5	54.2	0.03
MMSE at baseline,/30	29.1 (1.0)	27.0 (1.8)	23.4 (1.9)	<0.001
Length of follow-up, y Minimum, maximum	2.6 (0.8) 0.5, 3.7	2.3 (0.8) 0.5, 3.5	1.7 (0.6) 0.5, 3.1	<0.001
BSI measurements per subject, No. Minimum, maximum	3.2 (0.9) 1, 4	3.6 (1.3) 1, 5	2.3 (0.8) 1, 3	<0.001
Total brain volume, mL	1068 (102)	1059 (114)	1022 (115)	<0.001^a^
Total hippocampal volume, mL	5.2 (0.7)	4.5 (0.8)	3.9 (0.9)	<0.001^a^
Total intracranial volume, mL	1446 (135)	1466 (145)	1450 (163)	0.3
White-matter hyperintensity, mL log_2_ WMH, mL	0.22 (0.5) −2.39 (2.3)	0.28 (0.6) −2.07 (2.4)	0.40 (1.0) −1.37 (2.2)	<0.001^a^
Percentage of APOE e4 carriers	27	56	70	<0.001
Percentage of hypertension	43	50	52	0.1
Percentage of diabetes	6	7	6	0.8
Percentage of hypercholesteremia	26	30	36	0.1
Years of education	16.05 (2.86)	16.66 (3.00)	14.81 (3.09)	<0.001
Years since AD symptom onset	–	–	3.6 (2.6)	–

Values are mean (standard deviation) unless reported. White-matter hyperintensity values reported as median with interquartile range.

Key: AD, Alzheimer's disease; BSI, boundary shift integral; MCI, mild cognitive impairment; MMSE, Mini–Mental State Examination; TIV, total intracranial volume.

a Adjusted for TIV.

### 3.2 Longitudinal VBM results

#### 3.2.1 Group differences in age–atrophy relationship

**Fig. 2:**
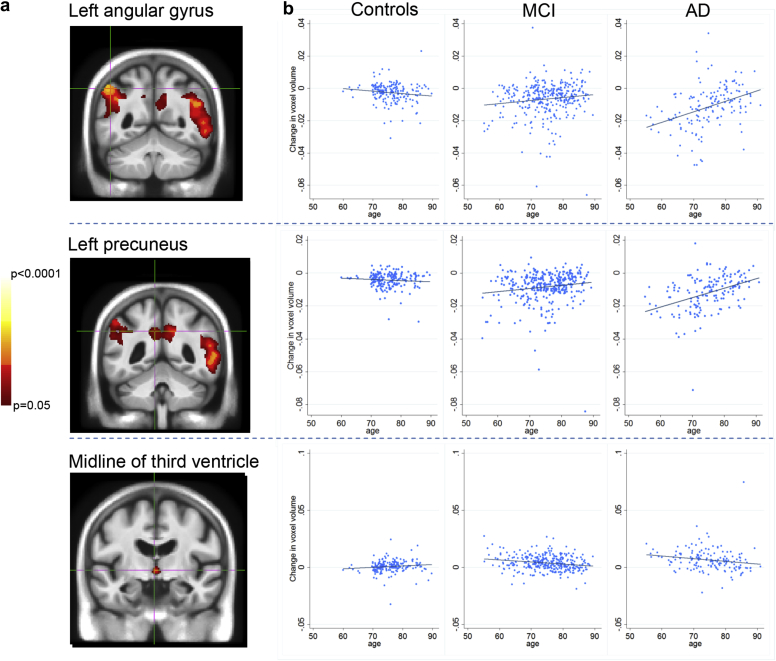
Results of the F test to test the age-by-group interaction term to predict volume change. (A) Clusters in the images represent voxels in which there is a significant difference in the relationship between age and atrophy rate across the 3 groups. (B) Graphs explain these relationships; summary slopes of voxel change for each individual at the voxel of interest are plotted against baseline age in controls, MCI, and AD patients. Positive values of change in voxel volume indicate expansion, and negative values represent voxel contraction. Results are adjusted for APOE genotype and white-matter hyperintensity volume. Each voxel of interest is located within an FWE corrected *p* < 0.05 cluster indicated by the crosshairs in the images. Abbreviations: AD, Alzheimer's disease; MCI, mild cognitive impairment.

Fig. 2A) shows the results of the F-test across all groups showing the interaction between age and atrophy. These results are FWE corrected, thresholded at *p* < 0.05 and adjusted for APOE and WMH. The precuneus, angular gyrus, superior temporal lobes, and midline of the third ventricle showed significantly different relationships between baseline age and volume change in each group. The graphs in Fig. 2B show the slope of volume change at a voxel plotted against baseline age within each cluster region (indicated by the crosshairs); this represents the effect of age in each group at that voxel. For AD patients, the left angular gyrus and precuneus showed a negative correlation between increasing baseline age and voxel contraction, indicating greater atrophy at younger ages in the voxel examined. This relationship was also seen in MCI, but to a lesser degree. For controls, there was little correlation between baseline age and atrophy, with a tendency toward greater contraction with increasing age. These relationships were seen bilaterally.

The cluster on the midline of the third ventricle is likely partial volume, reflecting differences in the effect of age on ventricular change rates. An increase in voxel expansion with age is seen in controls, as opposed to greater expansion at younger ages in AD subjects. The results for models without adjustment for WMH and APOE genotype are shown in Supplementary Material (Supplementary Fig. 1), as are all models for WM (Supplementary Fig. 2).

#### 3.2.2 Differences between controls and AD patients in age–atrophy relationship

**Fig. 3:**
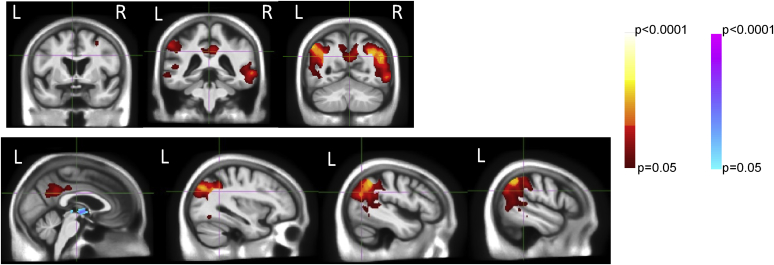
Results of the T tests to directly compare the age-by-group interaction between controls and AD patients. Clusters indicate regions in which the relationships between age and atrophy are different between groups, that is, differences in age-by-group interaction. Red clusters signify regions in which there is greater atrophy at younger ages in AD patients, whereas for controls, there is little age–atrophy relationship. Blue clusters indicate voxels which expand more at younger ages in AD patients, whereas controls expand more at older ages. There were no differences between control and MCI patients. Analyses are corrected for multiple comparisons, FWE *p* < 0.05, and are also corrected for APOE genotype and WMH volume. Abbreviations: AD, Alzheimer's disease; FWE, family-wise error; MCI, mild cognitive impairment; WMH, white-matter hyperintensity. (For interpretation of the references to color in this figure legend, the reader is referred to the Web version of this article.)

A T contrast between control and AD groups revealed an extensive posteromedial region with differential age relationships (see Fig. 3 for FWE corrected, APOE- and WMH-adjusted results). This area encompasses the bilateral precuneal, posterior cingulate, parietal, and superior temporal lobes. The left supramarginal gyrus, left fusiform, and right prefrontal cortex also demonstrate this relationship. A midline cluster is also present with the reverse contrast (visualized in blue in Fig. 3), this is likely to represent partial volume, reflecting differences between groups in ventricular expansion with age, as explained in Section 3.2.1 and illustrated in Fig. 2.

#### 3.2.3 Differences between controls and MCI patients in age–atrophy relationship

There were no significant differences in age–atrophy relationships between control and MCI patients.

#### 3.2.4 Age atrophy association without correction for APOE genotype and WMH

Results for the overall test of the age–atrophy interaction term, and for the comparison between controls and AD appear materially unchanged with the omission of APOE genotype and WMH as covariates, see Supplementary Fig. 1. Differences with and without APOE and WMH covariates (Fig. 2 and Supplementary Fig. 1) were not directly compared; therefore, visual differences between the 2 models should be interpreted with caution.

#### 3.2.5 Age–atrophy association in white matter

Strong differences in age and atrophy relationships between groups were also found in the white matter, with and without correction for WMH and APOE genotype, see Supplementary Fig. 2. The white matter subjacent to the GM in these subjects appears to be similarly affected by age; areas of white matter with differential age atrophy patterns across groups were found directly proximal to regions of GM exhibiting similar age effects as seen in equivalent earlier models (see Fig. 2 and Supplementary Fig. 2).

### 3.3 Longitudinal age atrophy rate associations

Table 2 shows the partial regression coefficients for the association between age and longitudinal brain and hippocampal change, adjusted for WMH and APOE genotype. AD patients had the highest atrophy rates of 14 mL/y (95% CI: 12.94–15.12), for the whole brain and 0.2 mL/y (0.16–0.20) for the hippocampus, MCI followed with rates of 10 mL/y (9.17–10.70) for the whole brain and 0.1 mL/y (0.11–0.14) for the hippocampus, for APOE negative individuals with the mean age, TIV and WMH load. Controls had an average atrophy rate of 6 mL/y (5.66–6.72) for the whole brain and 0.06 mL/y (0.05–0.07) for the hippocampus, for APOE negative individuals with the mean TIV and WMH load.

**Table 2:** Results from the regression model assessing the relationship between change in brain and hippocampal volume (left and right summed) and age by each diagnostic group (estimated using an age-by-diagnostic group interaction)

	Whole brain	Hippocampus
Atrophy rate (mL/y)		
Controls	6.19	0.06
	(5.66, 6.72)	(0.05, 0.07)
	(<0.001)	(<0.001)
MCI	9.93	0.13
	(9.17–10.70)	(0.11, 0.14)
	(<0.001)	(<0.001)
AD	14.02	0.18
	(12.94, 15.12)	(0.16, 0.20)
	(<0.001)	(<0.001)
Age interaction (mL/y/decade)^a^		
Controls	0.16	0.03
	(−0.79, 1.11)	(0.01, 0.04)
	(0.8)	(<0.001)
MCI^∗^	−1.90	−0.04
	(−3.19, −0.60)	(−0.06, −0.02)
	(0.003)^∗^	(<0.001)^∗^
AD^∗^	−2.64	−0.03
	(−4.25, −1.03)	(−0.05, −0.001)
	(0.001)^∗^	(0.04)^∗^

Average brain and hippocampal atrophy rates with *p* value and 95% confidence intervals are shown in mL/y.

For MCI and AD groups, age interaction estimates are given after subtraction of the estimate effect in controls (to account for normal aging), *p*-values for MCI and AD indicate whether the age–atrophy relationship is significantly different from controls (^∗^).

Key: MCI, mild cognitive impairment; AD, Alzheimer's disease.

a Age interaction estimates represent an increase in atrophy rate for a 10-year increase in baseline age (mL/y/decade), adjusted for total intracranial volume, APOE genotype, and WMH volume.

#### 3.3.1 Effect of age on atrophy rate in controls (normal aging)

We found that greater age at baseline was associated with significantly increased hippocampal atrophy rate (0.03 mL/y for a 10-year increase [0.01–0.04]) for controls, see Table 2; there was no evidence of an age effect on whole-brain volume change. The increase in hippocampal atrophy rate in controls was equivalent to an acceleration of 50% for a decade increase in age, for someone of average age at baseline (75 years).

#### 3.3.2 Differences between controls and AD patients in age–atrophy relationship

There were significant differences between age–atrophy relationships in controls and AD for whole-brain and hippocampal atrophy rates. Although there was no effect of age on whole-brain atrophy rate in controls, a 10-year increase in age from average was associated with a reduction in atrophy rate for AD patients of 3 mL/y (1.03–4.25) after adjusting for WMH and APOE e4 and subtracting the age–atrophy effect in controls. In contrast to controls, younger age was associated with greater hippocampal atrophy rate in AD patients, corresponding to a reduction in atrophy rate of 0.03 mL/y (0.05, 0.001) for a 10-year increase in age after adjusting for WMH and APOE e4 and subtracting the age–atrophy effect in controls. The reductions in atrophy rate for AD patients correspond to a decrease of 20% for the whole brain and 15% for the hippocampus, for a decade increase in age, for someone of average age at baseline (75 years).

#### 3.3.3 Differences between controls and MCI patients in age–atrophy relationship

There were also significant differences between age–atrophy relationships between controls and MCI for whole-brain and hippocampal atrophy rates. Similarly to AD patients, age was associated with a reduction in atrophy rate for MCI patients of 2 mL/y (0.60–3.19) for a 10-year increase in age after adjusting for WMH and APOE e4 and subtracting the age–atrophy effect in controls. Younger age was also associated with greater hippocampal atrophy rate in MCI, in which a reduction in atrophy rate of 0.04 mL/y (0.02–0.06) for a 10 year increase in age was seen after adjusting for WMH and APOE e4 and subtracting the age-atrophy effect in controls. For MCIs, the reduction in atrophy rate for a decade increase in age corresponds to 20% for the whole brain and 15% for the hippocampus, for someone of average age at baseline (75 years).

### 3.4 Further analyses

Posterior atrophy at younger ages in AD remained in a CSF subset of amyloid positive AD and MCI patients, compared with amyloid negative controls, although the extent of the effects was much reduced, see Supplementary Fig. 4. The CSF amyloid confirmed subset also experienced greater whole-brain atrophy at younger ages, see Supplementary Table 3.

There was no significant effect of age on baseline brain volume in AD, after adjustment for TIV and normal aging, see Supplementary Table 2.

In analyses investigating change in MMSE and age, younger AD patients were found to have faster decline in MMSE, whereas the opposite was found in controls, see Supplementary Table 4.

Results from models investigating the effect of age at onset on AD are shown in Supplementary Fig. 5 and Table 5. Similar effects compared with baseline age were found for VBM and BSI analyses.

Results from models investigating the linearity of the effect of age on atrophy rates are shown in Supplementary Table 6. No evidence of nonlinearity was found.

## 4 Discussion

In this large multisite study designed to mimic a clinical trial, we used a novel voxel-wise approach to demonstrate extensive progressive posterior atrophy patterns in AD at younger ages, which remain in a cohort with confirmed AD pathology using CSF data. We found that atrophy rate and patterns of atrophy varied differentially with age between cognitively normal, mildly impaired, and clinical AD patients. Younger AD subjects showed faster whole-brain and hippocampal atrophy rates and greater volume loss in association cortices with predominantly posterior and posteromedial regions affected compared to older AD patients. In contrast, the hippocampal atrophy rates were slower among younger controls, and controls had little other difference in atrophy with age. In MCI and AD patients, atrophy rate reduced with age. These findings were apparent after WMH and APOE e4 adjustment, suggesting that this difference is unlikely to be explained by small vessel disease or by APOE genotype. Given that results are adjusted for variables that have been associated with the aging process (WMH), and represent differences in slopes from normal controls, these differences in disease progression are likely to be driven by differences in age at onset in MCI and AD.

### 4.1 Effect of baseline age on atrophy patterns

We found that the bilateral posterior parietal, posterior cingulate, posterior temporal, and precuneal regions were more vulnerable to atrophy in younger AD patients. This is consistent with cross-sectional findings that younger AD subjects have less volume in association cortices (Aziz et al., 2017, Frisoni et al., 2005, Harper et al., 2017). In a small scale study, Cho et al. found similar regions affected more in early-onset AD versus late-onset AD (Cho et al., 2013a). The precuneus has been found to be involved in early-onset AD before by other studies (Karas et al., 2007, Möller et al., 2013), as have the parietal lobes (Frisoni et al., 2007, Holland et al., 2012, Holland et al., 2013), particularly at the temporoparietal junction (Frisoni et al., 2005). However, unlike Cho et al., we did not find the caudate, thalamus, or basal ganglia to be involved in patients at younger ages (Cho et al., 2013b). In addition, our results do not fit with a recent study by Knopman et al., who found early-onset patients showed greater deficits in glucose metabolism compared to late-onset patients but no differences in cerebral atrophy (Knopman et al., 2016). Knopman was an observational community-based study, whereas the present study is a mock clinical trial. The difference in findings may be due to the restricted usage of AD-signature regions of interest for the atrophy measures (entorhinal, inferior temporal, middle temporal, and fusiform gyrus) but a more inclusive set of ROIs for the ^18^F-fluorodeoxyglucose (FDG) positron emission tomography analyses (including the angular gyrus and posterior cingulate regions). These posterior regions have been found to be particularly vulnerable to atrophy in younger subjects, and others have also reported their metabolic vulnerability at younger ages (Kemp et al., 2003, Rabinovici et al., 2010). Our results suggest that if ROI approaches are used, then the ages of the subjects concerned require consideration; different ROIs may be needed for young-onset subjects to fully capture AD-related changes.

The association cortices were found to be more vulnerable at younger ages in AD, corresponding to previously reported cognitive deficits that are more often experienced by younger patients. Younger patients are more likely to first experience an impairment in judgment, problem solving, language, or visuospatial function than older patients (Barnes et al., 2015, Koedam et al., 2010). Early-onset AD patients are also more likely to perform poorly on tests of attention and frontoexecutive function (Cho et al., 2013a, Frisoni et al., 2007). Our study included a very small number of early-onset AD patients (n < 65 years = 17), symptoms described as occurring more frequently in younger patients correspond to parietal, precuneal, and frontal lobe atrophy patterns found in our study. Our results also show that younger AD patients have faster MMSE decline as well as faster atrophy rates compared with older patients (see Supplementary Table 4). Studies categorizing AD patients according to their patterns of atrophy find increased probability of posterior cortical involvement at younger ages (Na et al., 2016, Shiino et al., 2006, Whitwell et al., 2012). Na et al. also found parietal subtypes to have faster progression in multiple cognitive domains compared with cases with either medial temporal or diffuse atrophy patterns (Na et al., 2016). Younger patients are more likely to have diffuse cortical neurofibrillary tangles and sparing of the hippocampi at autopsy, whereas those with AD pathology in predominantly limbic areas are more likely to be older and of the APOE e4 genotype (Murray et al., 2011). Suarez-Gonzalez et al. have shown that even within an atypical cohort of patients with PCA, early-onset patients display atrophy in more posterior regions (Suárez-González et al., 2016).

As ADNI was designed to emulate a clinical trial, the presence of such diverse neuroimaging atrophy patterns within a trial population may be an important source of variability in trials where atrophy rates are used as outcome measures. Furthermore, although diminished, the phenotype of extrahippocampal atrophy at younger ages remained in a smaller subset of participants with confirmed underlying amyloid pathology, as measured by CSF amyloid beta 1–42 (see Supplementary Fig. 4, Table 3). Although early-onset patients are more likely to have faster rates of atrophy and provide greater power to detect a disease-modifying effect (Holland et al., 2012), the etiology underlying extrahippocampal atrophy patterns in younger patients may have an impact on drug effectiveness. More research is required to understand whether specific outcome measures that are appropriate for older populations (hippocampal rates and memory-weighted tests) are the optimal choice in clinical trials in younger patients. Trials may benefit from restricting samples to individuals with a typical pattern of atrophy as previously suggested (McEvoy et al., 2008, Yu et al., 2014) or by stratifying for atrophy pattern. In addition, variability in individuals' rates of atrophy in clinical trials can compromise detection of an overall group treatment effect. Usage of clinical trial run-in to assess each subject's initial rate of progression may provide greater power to detect a treatment effect when testing a trial population containing both younger and older AD subjects (Frost et al., 2008).

Here, we provide evidence that a posterior predominance in atrophy rate at younger ages is unlikely to be accounted for by APOE e4 genotype. Genetic differences have previously been used to explain differences in age at onset, clinical phenotype, and brain regions affected in AD. The effect of APOE e4 on age at onset is complex. Although APOE e4 has been found to accelerate disease onset (Breitner et al., 1998), it is thought to be less common in atypical early-onset AD (van der Flier et al., 2011). The APOE e4 allele has been found to be associated with typical AD; memory problems (Lehtovirta et al., 1996), targeted hippocampal atrophy (Manning et al., 2014), and greater burden of AD pathology present in the hippocampus at autopsy (Murray et al., 2011). Holland et al. found that the effect of atrophy slowing with age in AD was stronger in APOE e4 noncarriers than carriers in the entorhinal cortex; this corroborates the finding by Na et al., of faster cognitive decline in early-onset atypical AD cases, which trended toward absence of an APOE e4 allele (Holland et al., 2013, Na et al., 2016). However, the presence of an APOE e4 allele is a significant risk factor for PCA, a type of atypical early-onset AD; although the risk conferred by the gene for PCA is reduced compared with typical AD (Schott et al., 2015). In addition to APOE genotype, including WMH as a covariate in our study did not affect the results, indicating that the posterior patterns of atrophy at younger ages are unlikely to be driven by WMH or APOE genotype. This is important since WMH in AD subjects may also reflect pathologic AD processes, such as axon demyelination, Wallerian degeneration, or cerebral amyloid angiopathy (Ryan et al., 2015, Schmidt et al., 2011). Yet unknown genes and other factors may underlie the causes of extramedial temporal lobe atrophy, faster progression, and younger onset of clinical symptoms.

### 4.2 Effect of baseline age on summary rates of atrophy (whole brain and hippocampus)

In our BSI analyses, we found higher hippocampal atrophy rates with age in controls but did not find greater atrophy rates in older AD subjects. Contrastingly, we found the opposite effect in AD subjects compared to controls, in which a reduction in hippocampal atrophy rate with age was present, similarly to the study by Holland et al. (Holland et al., 2013). Others have found mixed results cross-sectionally, with some authors reporting smaller hippocampi in late-onset AD cases compared with early-onset cases (Frisoni et al., 2005, Frisoni et al., 2007, Möller et al., 2013), and another reporting similar levels of hippocampal atrophy across the AD age span compared to healthy controls (van de Pol et al., 2006). These mixed results in the literature may be due to differences in the way of correcting for normal aging and differing techniques used (volumetry vs. VBM). Cho et al. found in a longitudinal whole brain cortical thickness study, the parahippocampal gyrus exhibited more rapid thinning in late-onset AD compared to early-onset AD (Cho et al., 2013a); however, a further study of longitudinal volume loss in subcortical structures showed no difference in volume reduction in the hippocampus between early- and late-onset AD (Cho et al., 2013b). In a subset of ADNI including baseline and 12-month scans only, Evans et al. also investigated an age-by-group interaction on atrophy; finding higher age was associated with greater whole-brain atrophy and ventricular expansion in AD and the opposite effect in controls (Evans et al., 2010). Our AD subjects had significantly greater WMH volume despite being a similar age to controls. This may be due to greater small vessel disease in AD subjects, cerebral amyloid angiopathy, or a feature of advanced AD due to pathologic breakdown of white matter. As the same WMH effect was estimated across all groups, the presence of nonaging-related WMH in younger AD subjects may mean that the WMH effect in controls with age is not completely removed. For controls, this may explain why an age effect remained in the hippocampus even after controlling for WMH. Alternatively, other factors associated with age may drive atrophy in these subjects. The fact that the hippocampi were not found to be differentially affected by age in each group from VBM analyses may be due to limitations of voxel-based analyses in medial temporal lobe areas.

### 4.3 Strengths and limitations

This is the largest study investigating age and voxel-wise atrophy patterns in patients with MCI or AD to date, and the only study to consider the AD–age relationship in the context of APOE e4 genotype, presumed vascular pathology, and normal aging. Using age as a continuous variable, we did not split cases by arbitrary cutoffs, as has been done in previous studies where 65 years divide early- and late-onset. A major strength is that, using the multi-time point technique, we were able to include subjects which would have previously been excluded due to dropout, reducing bias compared to other studies in which dropouts tend to have more vascular pathology and smaller brain volumes (Fiford et al., 2017). Therefore, our results are unlikely to be driven by frail older subjects, who may have progressed more quickly, leaving the study; our statistical techniques were able to accommodate missing data by including all subjects with at least 1 follow-up scan. However, the number of young AD subjects was not large, and all controls were aged greater than 60 years (see Supplementary Table 1 for demographic data split by age in each group), this limits the generalizability of our findings, which require replication in a more age-balanced cohort. We additionally did not investigate the scatter plots of slopes of the age-by-group interaction in each voxel but have made the assumption that the relationship was similar across the different tissue types and regions affected; some voxels within each cluster may exhibit stronger or weaker effects, especially at their periphery. The homogeneous nature of white matter provides less intensity information for registration than gray matter; therefore, localized white-matter volume changes should be interpreted cautiously. The use of 1.5-T MRI may be considered a limitation; many studies are conducted using 3-T scanners. However, 1.5-T scanners are still used at many clinical centers including those which conduct research and clinical trials. The ROIs in this study have been limited to the whole brain and hippocampus, in line with metrics used in clinical trials. Future work in different datasets, investigating ROIs, including the posterior association cortices identified in this study, will allow assessment of the potential utility of these new ROIs in clinical trials.

Our study included a small number of AD patients aged less than 65 years (n = 17), and all patients had memory problems in line with a typical late-onset phenotype. Our findings may not apply to an exclusively early-onset population and require replication in a more age-balanced sample. Therefore, our results should be cautiously compared with the early-onset AD literature. Despite the inclusion criteria and demographics of our group, we were able to demonstrate that age influences atrophy rates and patterns of progressive atrophy in AD. Given that this cohort replicates that of a clinical trial, this has important ramifications for the choice of outcome measures in studies with a similar setup. Our results suggest a continuum of age-related differences across the age range of AD, beyond the cutoff of 65 years for early-onset AD cases. We also found no evidence of a nonlinear age relationship from voxel-wise and ROI analyses (see Supplementary Data, Table 6). However, as the data is imbalanced in terms of age of subjects, these results should be interpreted cautiously.

We have not fully investigated differences in disease severity with age in our study. However, we have found no evidence to suggest subjects were more severe or had longer symptom duration with age; the elapsed time since diagnosis and MMSE scores were similar in older and younger subjects, and they also remained in the study for similar lengths of time (see Supplementary Table 1). Similar disease severity is also suggested by the finding of no difference in baseline brain size of AD subjects with age (see Supplementary Table 2) after accounting for the effect of normal aging and TIV. Finally, analysis using the age of individuals at baseline appears to show similar results for age at symptom onset (as estimated by a study partner), (see Supplementary Fig. 5, Table 5).

## 5 Conclusions

Age is an important modifier of AD; younger amnestic AD patients display extensive posterior and medial-posterior atrophy, faster rates of atrophy, and cognitive decline compared with older AD patients. Notably, our results remain in a cohort with AD pathology confirmed using CSF amyloid beta 1–42 and demonstrate the influence of age in AD patients is not the effect of normal aging added to an AD atrophy signature. These distinct differences with age were found within AD patients selected to represent a clinical trial population and in those with CSF-confirmed amyloid pathology; therefore, our results have important implications for drug development. For clinical trials using atrophy rates as outcomes, younger subjects may provide more power to detect a treatment effect due to faster rates of atrophy; but they may also require revised outcome measures incorporating regions beyond the medial temporal lobe according to the average age of the population. Finally, an effective drug tested in a younger population may be of limited use in a general AD population if age effects on atrophy patterns represent differing underlying causes. More research is required to understand what drives tissue loss in different regions and at varying ages.

## Disclosure statement

GRR has received honoraria for teaching on SPM courses. JB has received honoraria for reviewing grants for the Fundação para a Ciência e Tecnologia, Portugal. None of the other authors have any conflicts of interest to declare.
