# Supplementary material for: Patterns of progressive atrophy vary with age in Alzheimer's disease patients
Source: Neurobiol Aging. 2018 Mar;63:22–32. doi: 10.1016/j.neurobiolaging.2017.11.002 (PMC5805840; doi:10.1016/j.neurobiolaging.2017.11.002)
Supplement: Supplementary Material [file mmc1.pdf]

Supplementary Material

Contents

Supplementary Methods 1
Demographics Split by Age 1
Grey Matter Volume change without correction for APOE e4 and WMH (model 2) 1
White Matter Volume change with correction for APOE e4 and WMH (model 3)........ 2
White Matter Volume change without correction for APOE e4 and WMH (model 4)........ 2
Regression of Baseline Brain Volume and Age 2
Analyses in CSF confirmed dataset........ 2
Relationships between age and cognition 2
Analyses using age at onset........ 3
Linearity analyses........ 3
Supplementary Results........ 4
Demographics table split by mean age........ 4
Differences in baseline brain volume with age........ 5
GM volume change; differences in age-diagnostic group interaction without correction for WMH or APOE genotype........ 6
WM volume change; differences in age-diagnostic group interaction........ 7
CSF confirmed results subject selection flowchart........ 8
CSF confirmed VBM results........ 9
CSF confirmed BSI results 10
Relationships between age and cognition 11
Age at onset VBM comparison 11
Age at onset BSI comparison 12
Linearity Relationships........ 13
References........ 14

Supplementary Methods

Demographics Split by Age

Each diagnostic group (control, MCI and AD) was split into two groups by the median age (75 years). For each age group the mean demographic factors were reported (see table 1).

Grey Matter Volume change without correction for APOE e4 and WMH (model 2)

GM volume change (outcome) was investigated with a main effect of group, a linear interaction term between group and baseline age, and with TIV as a covariate. See main paper for more information.

White Matter Volume change with correction for APOE e4 and WMH (model 3)

WM volume change was also investigated, with a main effect of group, a linear interaction term between group and baseline age, and with WMH, APOE e4 allele presence and TIV as a covariate. See main paper for more information.

White Matter Volume change without correction for APOE e4 and WMH (model 4)

WM volume change was also investigated, with a main effect of group, a linear interaction term between group and baseline age, and TIV as a covariate. See main paper for more information.

Regression of Baseline Brain Volume and Age

Brain volumes were estimated from the 1.5T volumetric T1-weighted images using a multi atlas template brain segmentation method (Leung et al., 2011).

To analyse the cross-sectional relationship between baseline brain volume and age at baseline a multiple linear regression was performed with baseline brain volume as the outcome, age as the predictor variable, and diagnosis and TIV as covariates. See supplementary table 2 for results.

Analyses in CSF confirmed dataset

CSF data was collected in a subset of ADNI participants (n=352), using a previously described method (Shaw et al., 2009). We classified patients by amyloid status using the abeta 1-42 cut off of 192pg/ml (Shaw et al., 2009); controls were selected for analysis if they were abeta negative (>192pg/ml) and MCI and AD patients selected if abeta positive (<192pg/ml). The final subset for analysis was 270 participants (see supplementary figure 3).

VBM steps were completed as per the pipeline described in the main paper section 2.2.1 until the DARTEL stage, in which the DARTEL registration was limited to the subset with CSF data (n= 270). The volume change maps of the subset were aligned to this CSF specific template.

The main model of GM change with age as a predictor was run in this CSF subset, WMH, TIV and APOE status (presence/absence of an e4 allele) were used as covariates, see the main paper for more details regarding the model (model 1, section 2.2.1). Models of BSI in this subset were also fitted in the CSF subset, as in main paper (section 2.2.2).

Relationships between age and cognition

We fitted multilevel linear mixed-effects regression models for repeated measures of MMSE (Frost et al., 2004). Interval in years between baseline and follow up was included as a fixed effect, in order that the resulting coefficient represented change in MMSE per year (outcome). The following covariates were included as main effects and as interaction terms with interval, in order that their inclusion could affect mean MMSE and how this changed over time: diagnostic group, an interaction between baseline age and diagnostic group, WMH, and APOE e4 carrier status (presence/absence of an e4 allele). Participant-level random effects for intercept and time since baseline MMSE measurement were included to permit between-participant heterogeneity in baseline MMSE and in rate of change in MMSE. Different random intercept and slope terms were fitted for control, MCI and AD groups, as the variability in MMSE is often higher in AD patients. In MCI and AD groups unstructured covariance of the random effects was used to allow for a correlation between baseline MMSE and rate of change in MMSE. A separate residual variance was fitted for each diagnostic group.

After estimation, the difference in the age effect on MMSE (baseline and change) for controls was subtracted from that in MCI/AD in order to account for normal aging.

Analyses using age at onset

For AD patients, age at symptom onset was calculated by subtracting the year of estimated symptom onset from the date of the baseline visit, in order to give years since AD onset. Year of estimated symptom onset was estimated by the study partner, the individual accompanying the patient to visits, with 10 hours or more contact per week with the patient. Years since AD onset was then subtracted from the patient's baseline age to give age at onset.

Relationships between baseline age and atrophy rate, or age at onset and atrophy rate, were run in ADs only (as there is no equivalent for variable for age at onset for controls). Four AD participants were excluded as there was no year of AD onset information. Analyses were run to estimate change in GM, predicted by either age at baseline or age-at-onset, see main paper section 2.2.1 model 1, adjusted for APOE (presence/absence of an e4 allele), WMH and TIV and for multiple comparisons after bootstrapping, family wise error correction ($p<0.05$). Results using age at onset and results using baseline age were qualitatively compared.

Linearity analyses

To examine the concept of non-linearity in the age relationship a quadratic term was added to the models of GM change predicted by age for BSI and VBM (age*age*diagnostic group), (main paper model 1, section 2.2.1). Models were adjusted for WMH, age and TIV. The outcome of the non-linear term investigated whether the effect of age on atrophy rates differed for a 10 year increase in age.

Supplementary Results

	Controls		MCI		AD	
	75 and under	Over 75	75 and under	Over 75	75 and under	Over 75
**N**	91	100	163	176	74	79
**Age at baseline, years**	71.7 (2.7)	79.7 (3.6)	68.9 (4.8)	80.7 (3.4)	68.5 (4.9)	81.0 (3.9)
**Percentage male**	53	51	59	66	54	54
**MMSE at baseline, /30**	29.1 (1.1)	29.2 (0.9)	27.1 (1.8)	26.8 (1.7)	23.6 (1.8)	23.2 (2.0)
**Length of follow up, years**	2.6 (0.7)	2.6 (0.8)	2.3 (0.8)	2.3 (0.8)	1.6 (0.64)	1.7 (0.60)
**Min., max. length of follow up, years**	0.5, 3.4	0.5, 3.7	0.5, 3.5	0.5, 3.4	0.5, 2.6	0.5, 3.0
**BSI measurements per subject, No.,**	3.3 (0.8)	3.2 (1.0)	3.6 (1.3)	3.5 (1.3)	2.2 (0.8)	2.4 (0.7)
**Total brain volume, ml**	1084 (106)	1054 (97)	1096 (111)	1025 (107)	1038 (123)	1009 (105)
**Total hippocampal volume, ml**	5.41 (0.73)	5.00 (0.65)	4.71 (0.85)	4.22 (0.77)	4.14 (0.92)	3.72 (0.83)
**Total intracranial volume, ml**	1446 (127)	1446 (142)	1481 (141)	1451 (148)	1443 (176)	1455 (151)
**White Matter Hyperintensity, ml**	0.17 (0.4)	0.25 (0.5)	0.17 (0.4)	0.40 (0.7)	0.31 (0.6)	0.54 (1.1)
**$\log_2$WMH, ml**	-2.75 (2.4)	-2.06 (2.2)	-2.73 (2.4)	-1.50 (2.1)	-1.71 (2.3)	-1.07 (2.1)
**Percentage APOE e4 carriers**	25	29	66	47	76	65
**Percentage Hypertensive**	36	49	47	53	45	58
**Percentage Diabetic**	5	6	9	5	3	9
**Percentage Hypercholesteraemic**	29	23	29	31	32	39
**Years of education**	15.7 (2.5)	16.3 (3.13)	15.8 (3.0)	15.6 (3.0)	14.7 (3.3)	14.9 (2.9)
**Years since AD symptom onset**	N/A	N/A	N/A	N/A	3.4 (2.4)	3.7 (2.7)

Supplementary Table 1: Subject demographics and basic imaging information, split by mean age (75 years) in each diagnostic group. Values are mean (SD) unless reported. Years since AD symptom onset are not-applicable for controls and MCI, as these subjects have not received an AD diagnosis.

	Baseline Brain Volume
Mean Brain volume adjusted for TIV, ml (constant)	1,078.4<br>[1071.0, 1085.8]
Effect of MCI[a]	-25.6<br>(<0.001)<br>[-34.8, -16.3]
Effect of AD[b]	-52.0<br>(<0.001)<br>[-63.1, -40.9]
Effect of age[c]	-37.7<br>(<0.001)<br>[-52.0, -23.4]
Effect of age in MCI (difference from effect of age controls) [d]	-1.29<br>(0.88)<br>[-17.5, 14.9]
Effect of age in AD (difference from effect of age in controls)[e]	7.00<br>(0.44)<br>[-11.0, 25.0]

Supplementary Table 2: Results from the regression model assessing the relationship between cross-sectional brain volume and age. Estimates are shown for difference in brain volume (ml) with (p values) and [95% confidence intervals] for, a diagnosis of MCI[a] or AD[b], and an increase in age of 10 years from the mean age[c], conditional on intracranial volume. The effect of age[c] is the effect of age in controls (normal aging), the effect of age in MCI[d] and AD[e] are differences from the effect in controls.

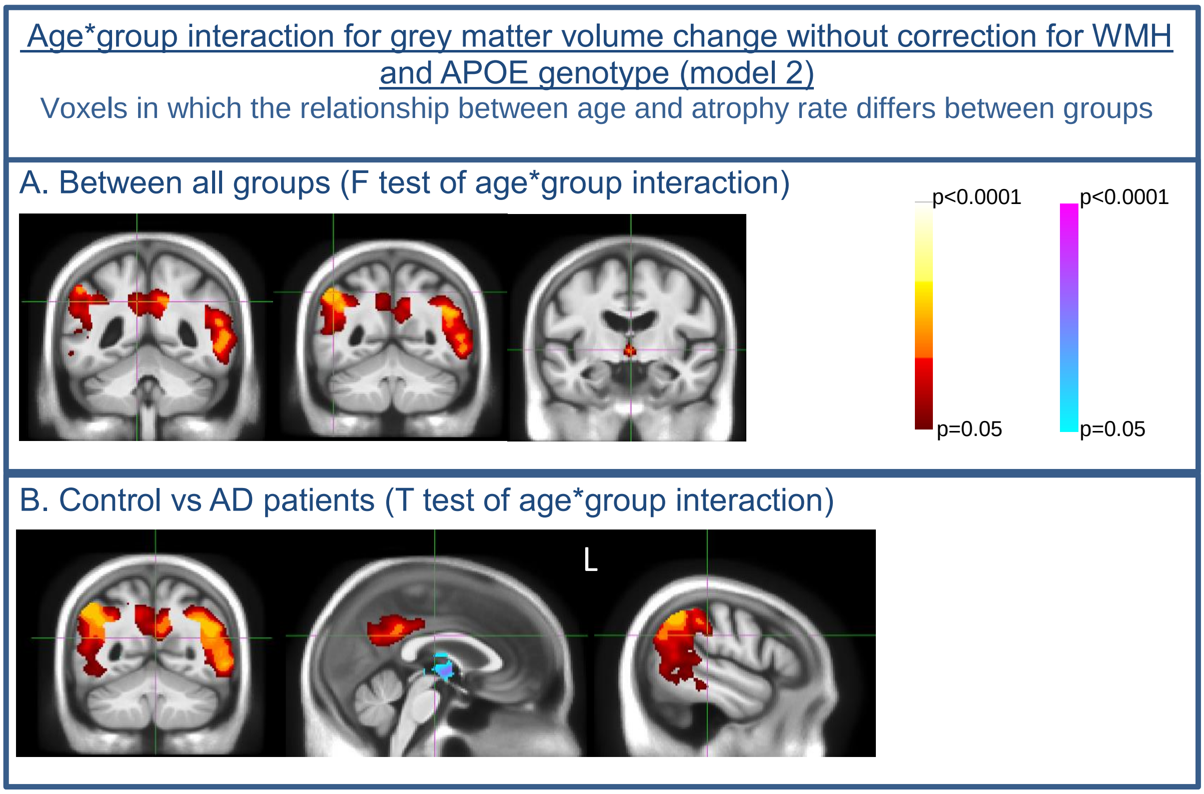

Supplementary Figure 1: Results of the F Test to test the age-group interaction term to predict volume change (A). Clusters in the images represent voxels in which there is a significant difference in the relationship between age and atrophy rate across the three groups. Results of the T tests to directly compare the age*group interaction between controls and AD patients (B). Clusters indicate regions in which the relationships between age and atrophy are different between groups. Red clusters in the control vs AD comparison signify regions in which there is greater atrophy at younger ages in ADs, whilst for controls there is little age-atrophy relationship. Blue clusters indicate voxels which expand more at younger ages in ADs, whilst controls expand more at older ages. There were no differences between control and MCI patients. Analyses are corrected for multiple comparisons, FWE $p<0.05$, and TIV.

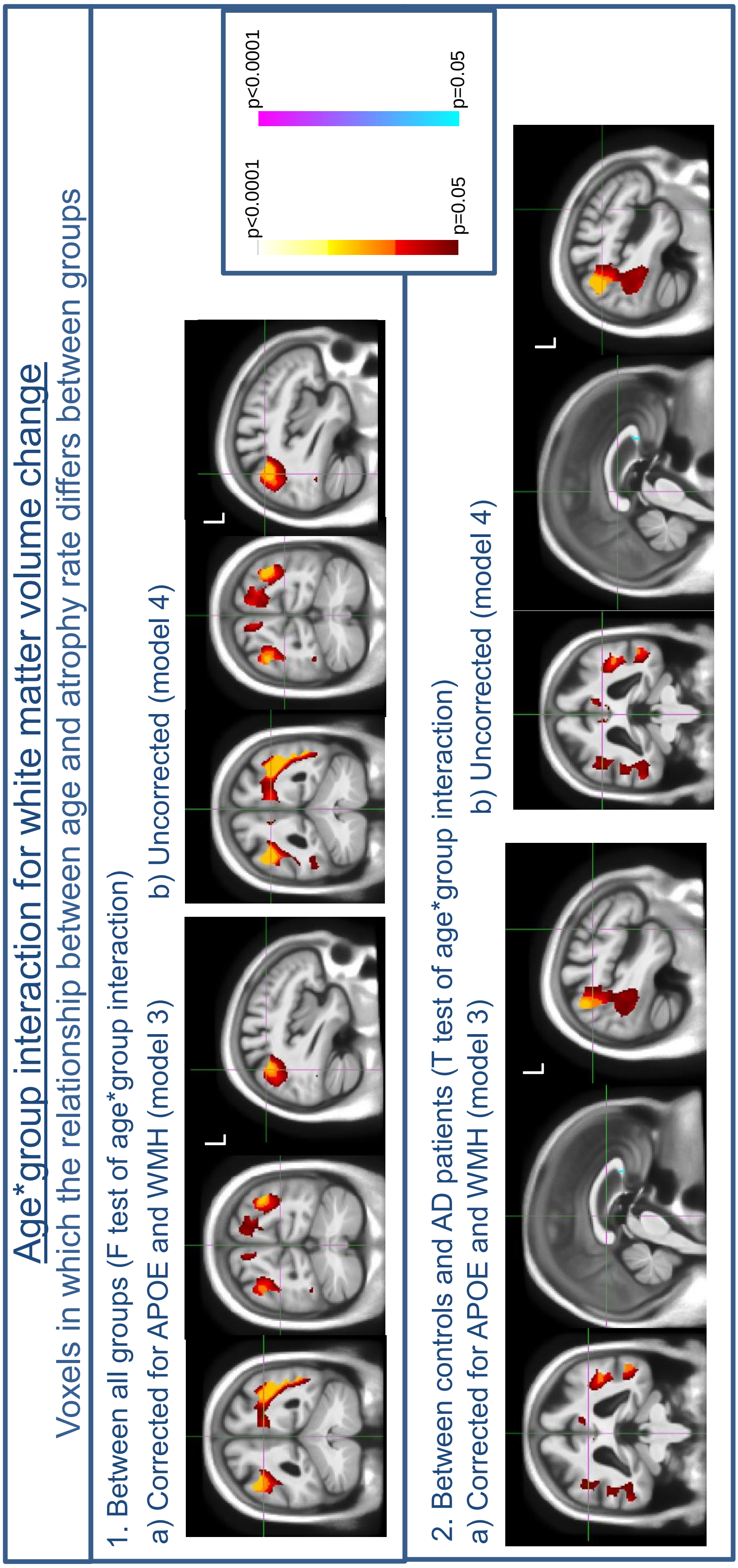

Supplementary Figure 2: Results of the F Test to test the age-group interaction term to predict white matter volume change corrected for WMH and APOE genotype (presence/absence of an APOE e4 allele) (1a, model 3), and uncorrected (1b, model 4). Clusters in the images represent voxels in which there is a significant difference in the relationship between age and atrophy rate across the three groups. Results of the T tests to directly compare atrophy-age relationship between controls and AD patients corrected for APOE and WMH (2a, model 3) and uncorrected (2b, model 4). Clusters indicate regions in which the relationships between age and atrophy are different between groups. There were no differences between control and MCI patients. All analyses are corrected for multiple comparisons,

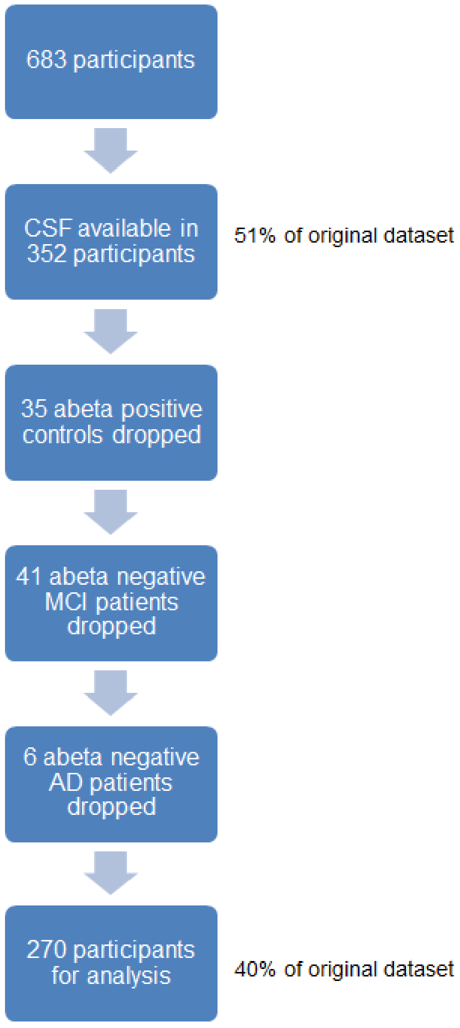

Supplementary Figure 3; Flowchart showing the selection of subjects for analysis based on CSF abeta 1-42 status.

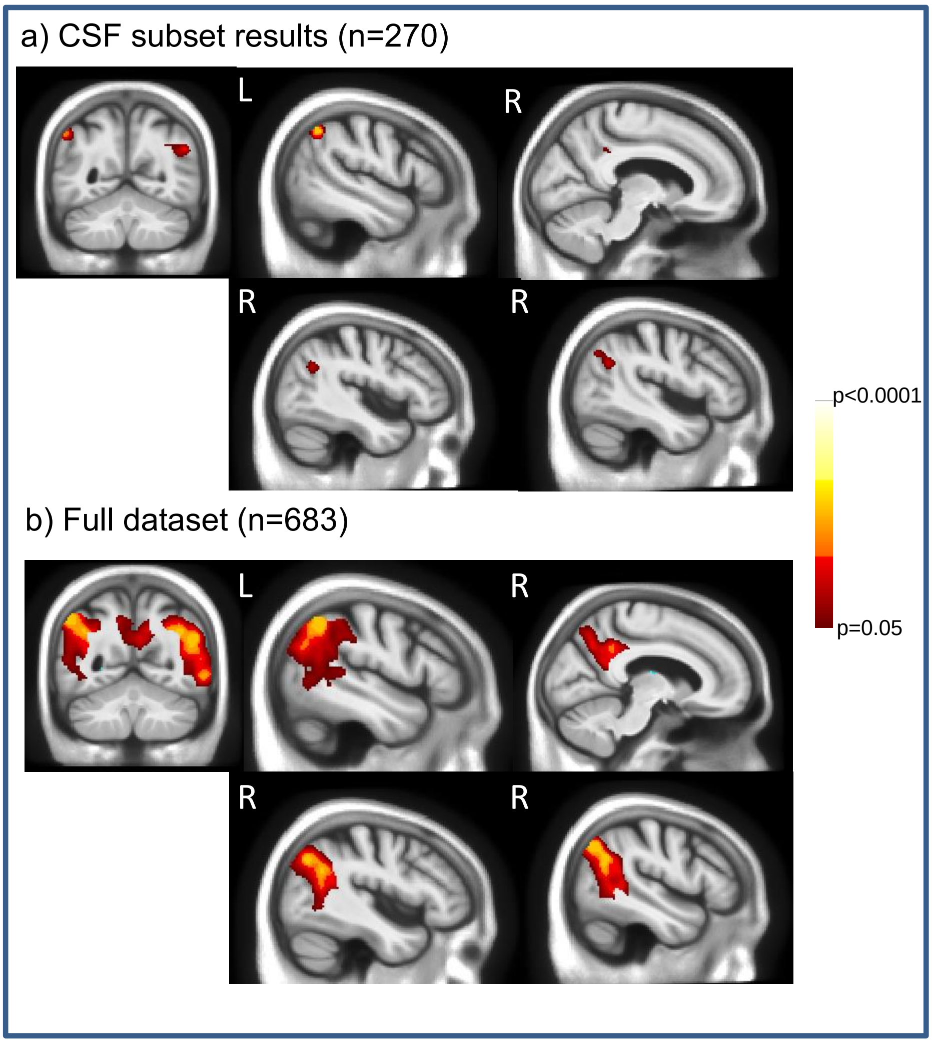

Supplementary Figure 4: Results of an age-by-group interaction between controls and AD patients in the subset with confirmed abeta 1-42 status (no amyloid pathology in controls and confirmed amyloid pathology in AD) (a) and the full dataset (b).Clusters indicate regions in which the relationships between age and atrophy are different between groups, i.e. differences in age-by-group interaction. Red clusters signify regions in which there is greater atrophy at younger ages in AD patients, whilst for controls there is little age-atrophy relationship. Analyses are corrected for multiple comparisons, FWE p<0.05, and are also corrected for APOE genotype (presence/absence of an e4 allele), TIV and WMH volume.

		Whole Brain	Hippocampus
**Atrophy Rate (ml/year)**	Controls	5.55<br>(<0.001)<br>[4.88, 6.21]	0.05<br>(<0.001)<br>[0.04, 0.06]
	MCI	10.94<br>(<0.001)<br>[9.64, 12.23]	0.15<br>(<0.001)<br>[0.13, 0.17]
	AD	13.00<br>(<0.001)<br>[11.35, 14.65]	0.17<br>(<0.001)<br>[0.14, 0.19]
**Age Interaction[a] (ml/year/decade)**	Controls	-0.433<br>(0.48)<br>[-1.639, 0.773]	0.023<br>(0.02)<br>[0.00, 0.04]
	MCI*	-1.79<br>(0.05)<br>[-3.57, -0.01]	-0.04<br>(0.003)<br>[-0.07, -0.02]
	AD*	-2.21<br>(0.04)<br>[-4.29, -0.13]	-0.004<br>(0.78)<br>[-0.03, 0.03]

Supplementary Table 3: Results from the regression model assessing the relationship between change in brain and hippocampal volume (left and right summed) and age by each diagnostic group (estimated using an age-by-diagnostic group interaction) in the subset with abeta status confirmed by CSF. Average brain and hippocampal atrophy rates with (p value) and 95% confidence intervals [95% CI] are shown in ml/year. Age interaction estimates (a) represent an increase in atrophy rate for a ten year increase in baseline age (ml/year/decade), adjusted for total intracranial volume, APOE genotype (presence/absence of an e4 allele) and WMH volume. For MCI and AD groups, age interaction estimates are given after subtraction of the estimate effect in controls (to account for normal aging), p values for MCI and AD indicate whether the age-atrophy relationship is significantly different from controls (*).

	Controls	MCI	AD
**Baseline MMSE**	29.06 (<0.001) [28.85, 29.28]	27.07 (<0.001) [26.80, 27.34]	23.45 (<0.001) [23.11, 23.89]
**Change in MMSE**	0.02 (<0.001) [-0.06, 0.10]	-1.04 (<0.001) [-1.24, -0.84]	-2.37 (<0.001) [-2.822, -1.911]
**Age effect on baseline MMSE**	-0.08 (0.62) [-0.39, 0.23]	-0.13 (0.54) [-0.56, 0.29]	-0.09 (0.7) [-0.62, 0.44]
**Age effect on MMSE change**	-0.16 (0.03) [-0.30, -0.02]	0.20 (0.18) [-0.09, 0.50]	1.24 (<0.001) [0.63, 1.85]

Supplementary Table 4: Results from a regression model investigating the effect of baseline age on change in MMSE (outcome). Estimates are shown for an increase in atrophy rate (ml/year) with (p values) and [95% confidence intervals] for a 10 year increase in age. Models are adjusted for total intracranial volume, APOE status (presence/absence of an e4 allele) and WMH.

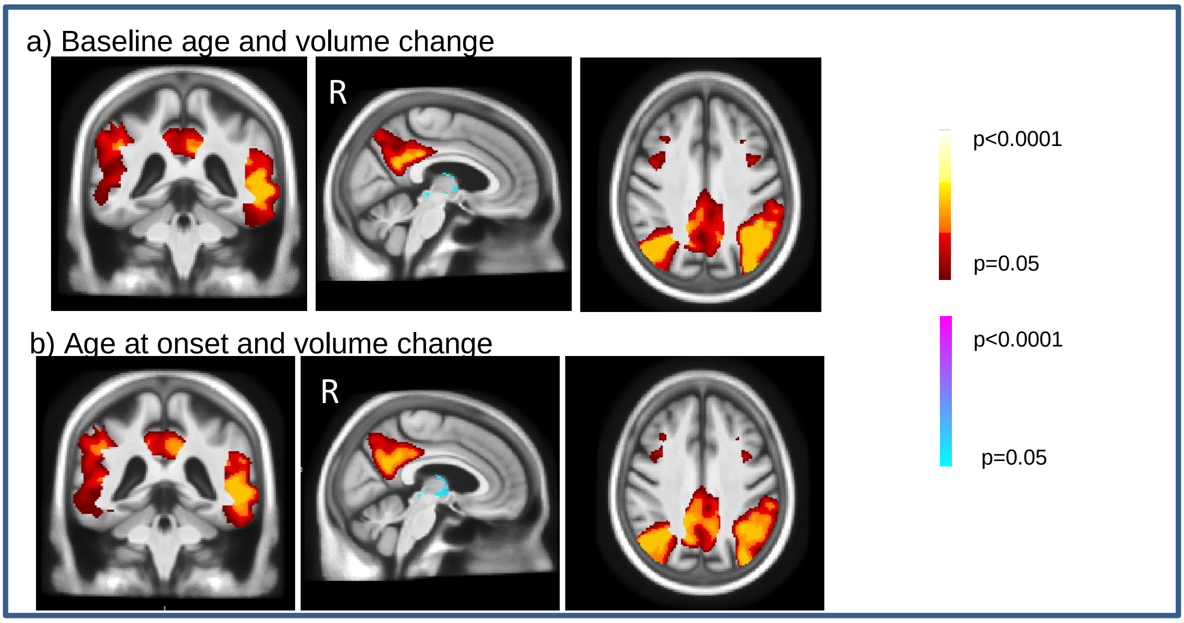

Supplementary Figure 5: VBM results showing the effect of age on GM change in AD patients using the variable baseline age (a), or age at onset (b). Red clusters signify regions in which there is greater atrophy at younger ages in AD patients, blue clusters indicate regions which expand more at younger ages. Analyses are corrected for multiple comparisons, FWE p<0.05 and are additionally corrected for presence or absence of an APOE e4 allele, TIV and WMH volume.

AD		Whole Brain	Hippocampus
**Atrophy Rate (ml/year)**	Baseline age	14.00 (<0.001) [12.22, 15.78]	0.17 (<0.001) [0.14, 0.20]
	Age at onset	13.90 (<0.001) [12.08, 15.70]	0.17 (<0.001) [0.14, 0.20]
**Age effect (ml/year/decade)**	Baseline age	-2.54 (<0.001) [-3.88, -1.19]	0.007 (0.51) [-0.01, 0.03]
	Age at onset	-2.00 (<0.001) [-3.17, -0.62]	0.011 (0.26) [-0.01, 0.03]

Supplementary Table 5: Results from a regression model investigating the effect of baseline age or age at onset on atrophy rates (outcome) in AD patients. Estimates are shown for an increase in atrophy rate (ml/year) with (p values) and [95% confidence intervals] for a 10 year increase in age. Models are adjusted for total intracranial volume, presence/absence of an APOE e4 allele and WMH.

		Whole Brain	Hippocampus
Atrophy Rate (ml/year)	Controls	6.23<br>(<0.001)<br>[5.65, 6.81]	0.06<br>(<0.001)<br>[0.05, 0.07]
	MCI	9.98<br>(<0.001)<br>[9.12, 10.82]	0.13<br>(<0.001)<br>[0.011, 0.14]
	AD	14.24<br>(<0.001)<br>[12.98, 15.50]	0.19<br>(<0.001)<br>[0.17, 0.21]
Age Interaction[a] (ml/year/decade)	Controls	0.19<br>(0.70)<br>[-0.78, 1.16]	0.03<br>(p<0.001)<br>[0.013, 0.045]
	MCI	-1.76<br>(<0.001)<br>[-2.70, -0.82]	-0.01<br>(0.15)<br>[-0.026, 0.004]
	AD	-2.59<br>(<0.001)<br>[-3.92, -1.25]	-0.001<br>(0.93)<br>[-0.022, 0.020]
Age quadratic term[b] (ml/year/decade)	Controls	-0.06<br>(0.8)<br>[-0.43, 0.32]	-0.002<br>(0.6)<br>[-0.007, 0.004]
	MCI	-0.02<br>(0.9)<br>[-0.29, 0.25]	0.000<br>(0.6)<br>[-0.004, 0.005]
	AD	-0.21<br>(0.7)<br>[-0.82, 0.41]	-0.01<br>(0.5)<br>[-0.016, 0.004]

Supplementary Table 6: Results from a regression model investigating a non-linear effect of age (predictor) on atrophy rates (outcome). Estimates are shown for an increase in atrophy rate (ml/year), (p value), and [95% confidence intervals]: a 10 year increase in age (constant age effect)[a], for a change in slope of the age-atrophy rate relationship for a 10 year increase in baseline age (quadratic age effect)[b]. Models are adjusted for age, total intracranial volume, presence/absence of an APOE e4 allele and WMH.

References

Frost, C., Kenward, M.G., Fox, N.C., 2004. The analysis of repeated “direct” measures of change illustrated with an application in longitudinal imaging. Stat. Med. 23, 3275–3286. doi:10.1002/sim.1909

Leung, K.K., Barnes, J., Modat, M., Ridgway, G.R., Bartlett, J.W., Fox, N.C., Ourselin, S., 2011. Brain MAPS: An automated, accurate and robust brain extraction technique using a template library. Neuroimage 55, 1091–1108. doi:10.1016/j.neuroimage.2010.12.067

Shaw, L.M., Vanderstichele, H., Knapik-czajka, M., Clark, C.M., Aisen, P.S., Petersen, R.C., Blennow, K., Soares, H., Simon, A., Lewczuk, P., Dean, R., Siemers, E., Potter, W., Lee, V.M., Trojanowski, J.Q., 2009. Cerebrospinal Fluid Biomarker Signature in Alzheimer’s Disease Neuroimaging Initiative Subjects. Ann Neurol 65, 403–413. doi:10.1002/ana.21610.Cerebrospinal
